# Escherichia coli ST2797 Is Abundant in Wastewater and Might Be a Novel Emerging Extended-Spectrum Beta-Lactamase E. coli

**DOI:** 10.1128/spectrum.04486-22

**Published:** 2023-06-01

**Authors:** Erik Paulshus, Patricia Colque, Inger Kühn, Tamanna Tauhid, Yue O. O. Hu, Yingshun Zhou, Kaisa Thorell, Roland Möllby, Henning Sørum, Åsa Sjöling, Enrique Joffré

**Affiliations:** a Department of Food Safety and Infection Biology, Faculty of Veterinary Medicine, Norwegian University of Life Sciences, Oslo, Norway; b Department of Analysis and Diagnostics, Norwegian Veterinary Institute, Ås, Norway; c Department of Microbiology, Tumor and Cell Biology (MTC), Karolinska Institutet, Stockholm, Sweden; d Centre for Translational Microbiome Research (CTMR), Karolinska Institutet, Stockholm, Sweden; e Department of Pathogen Biology, Southwest Medical University, Luzhou, Sichuan, China; f Department of Chemistry and Molecular Biology, University of Gothenburg, Gothenburg, Sweden; University of Arkansas for Medical Sciences

**Keywords:** ST2797, urban wastewater, multidrug resistant, persistent clones, PhenePlate, *Escherichia coli*, bacterial survival, biochemical fingerprinting, biofilm, genomics, multidrug resistant bacteria

## Abstract

The increasing prevalence of antibiotic-resistant bacteria is an emerging threat to global health. The analysis of antibiotic-resistant enterobacteria in wastewater can indicate the prevalence and spread of certain clonal groups of multiresistant bacteria. In a previous study of Escherichia coli that were isolated from a pump station in Norway over 15 months, we found a recurring E. coli clone that was resistant to trimethoprim, ampicillin, and tetracycline in 201 of 3,123 analyzed isolates (6.1%). 11 representative isolates were subjected to whole-genome sequencing and were found to belong to the MLST ST2797 E. coli clone with plasmids carrying resistance genes, including *bla*_TEM-1B_, *sul2*, *dfrA7*, and *tetB.* A phenotypic comparison of the ST2797 isolates with the uropathogenic ST131 and ST648 that were repeatedly identified in the same wastewater samples revealed that the ST2797 isolates exhibited a comparable capacity for temporal survival in wastewater, greater biofilm formation, and similar potential for the colonization of mammalian epithelial cells. ST2797 has been isolated from humans and has been found to carry extended spectrum β-lactamase (ESBL) genes in other studies, suggesting that this clonal type is an emerging ESBL E. coli. Collectively, these findings show that ST2797 was more ubiquitous in the studied wastewater than were the infamous ST131 and ST648 and that ST2797 may have similar abilities to survive in the environment and cause infections in humans.

**IMPORTANCE** The incidence of drug-resistant bacteria found in the environment is increasing together with the levels of antibiotic-resistant bacteria that cause infections. The COVID-19 pandemic has shed new light on the importance of monitoring emerging threats and finding early warning systems. Therefore, to mitigate the antimicrobial resistance burden, the monitoring and early identification of antibiotic-resistant bacteria in hot spots, such as wastewater treatment plants, are required to combat the occurrence and spread of antibiotic-resistant bacteria. Here, we applied a PhenePlate system as a phenotypic screening method for genomic surveillance and discovered a dominant and persistent E. coli clone ST2797 with a multidrug resistance pattern and equivalent phenotypic characteristics to those of the major pandemic lineages, namely, ST131 and ST648, which frequently carry ESBL genes. This study highlights the continuous surveillance and report of multidrug resistant bacteria with the potential to spread in One Health settings.

## OBSERVATION

**FIG 1:**
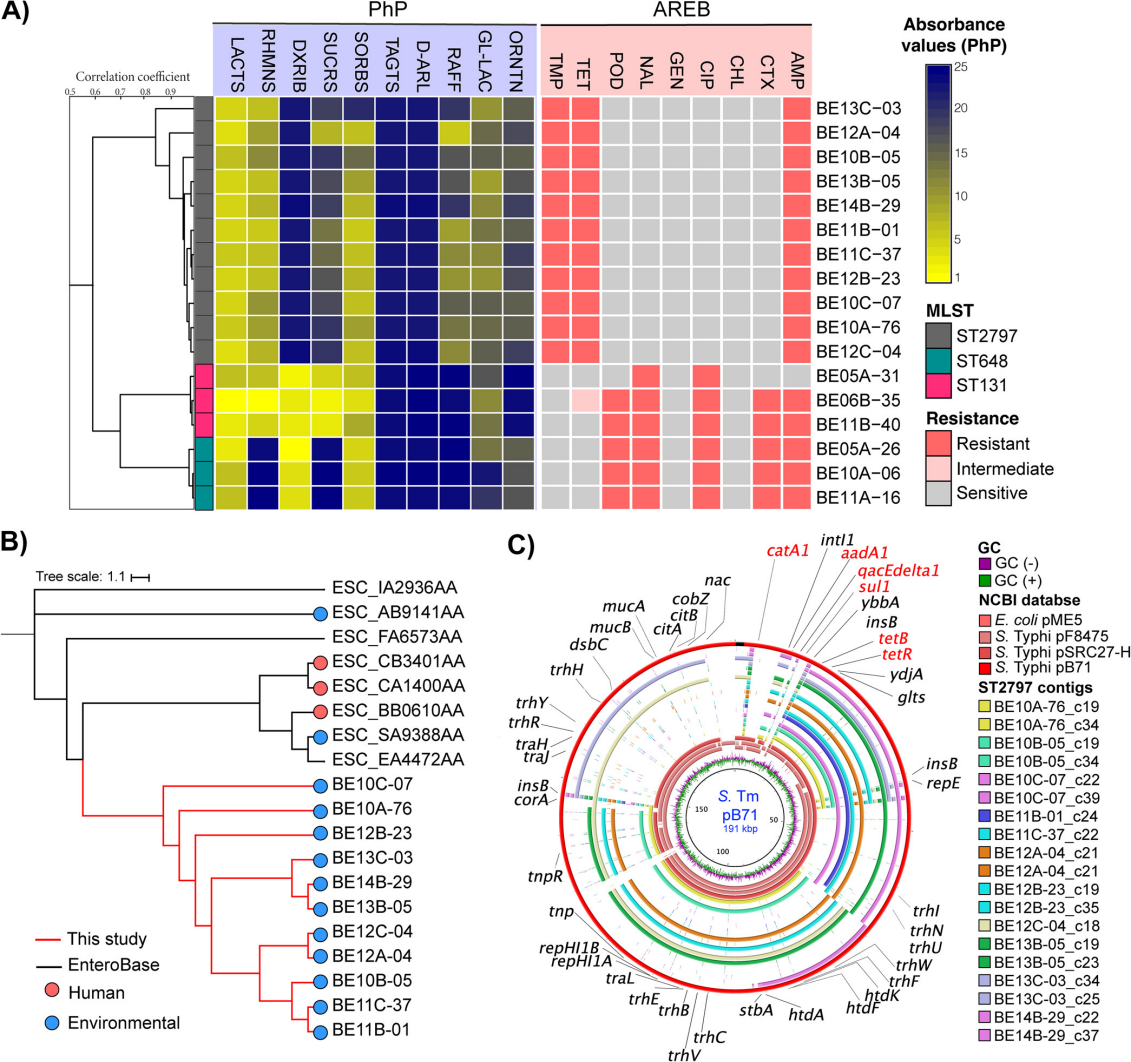
Comparison of the PhP-AREB profiles of the dominant clones found in urban wastewater. (A) Dendrogram inferred from the unweighted pair-group sequential clustering method using arithmetic averages (UPGMA) of the bacterial fingerprint of representative, whole-genome sequenced isolates of ST131 (PhP_1_), ST648 (PhP_2_), and ST2797 (PhP_3_). The absorbance profiles of sugar fermentation by bacterial isolates and the dendrogram were generated using the PhenePlate software package (PhPlate AB). The absorbance values ranged from positive or 0 (bright yellow) to negative or 25 (dark blue). The AREB plates were read as 0 (susceptible, growth ≤ 10% of the control well), 1 (intermediate, requiring visual inspection, growth = 10 to 25% of the control well), and 2 (resistant, growth ≥ 25% of the control well). The pairwise similarities among the isolates that were obtained from the data generated via the PhP method were expressed as correlation coefficients. LACTS, lactose; RHMNS, rhamnose; DXRIB, deoxyribose; SUCRS, sucrose; SORBS, sorbose; TAGTS, tagatose; d-ARL, d-arabitol; RAFF, raffinose; GL-LAC, gal-lactone; ORNTN, ornithine. The final concentrations (in mg/L) of antibiotics were: ampicillin (32), cefotaxime (2), chloramphenicol (32), ciprofloxacin (4), gentamicin (16), nalidixic acid (32), cefpodoxime (3), tetracycline (16) and trimethoprim (16). The PhP-AREB images of each plate were produced using a desktop scanner (HP G4050). (B) The SNP-based maximum likelihood dendrogram from 19 ST2797 isolates, of which 11 were obtained in this study and 8 were downloaded from EnteroBase (8) from multiple sources. The core genome alignment was generated by using the Panaroo pipeline (24) in the strict mode, a core threshold of 95%, and a maximum length difference between genes in a cluster of 75%, and it was composed of 4,054 genes. The tree was calculated using PhyML (25) with the standard parameters and SH-like branch supports. (C) The top five plasmid genomes with the highest percentage of identity (>70%) and query coverage were included in the comparison using BRIG36 (v0.95, http://brig.sourceforge.net/). Contigs that were longer than 10 kb from the ST2797 genomes and contained at least one antibiotic resistance gene were used as the reference sequences (outer rings). The inner rings represent GC Skew− (purple), GC Skew+ (green), E. coli strain EC 528 plasmid pME5 (MT868879.1), S. enterica subsp. enterica serovar Typhimurium strain B71 plasmid pB71 (KP899806.1), S. enterica subsp. enterica serovar Typhimurium strain F8475 plasmid pF8475 (KP899804.1), and S. enterica strain SRC27 plasmid pSRC27-H (CP058810.1). The outermost ring indicates the plasmid pB71-191kp with annotated genes.

Escherichia coli wastewater treatment plants (WWTPs) are considered to be hot spots for emerging clones of antibiotic resistant bacteria (1, 2). Untreated wastewater from urban WWTPs can be used as an early warning system for the emergence of new types of drug-resistant bacteria or viruses. We previously reported that a sample set of 3,123 E. coli isolates that were collected in Norway over 15 months from urban wastewater without hospital or other institutional affluents contained high levels of the emerging multiresistant clones ST131 (2.3%) and ST648 (3.8%) (2, 3). All of the isolates were initially phenotyped, and antibiotic resistance was tested using a PhenePlate-AREB system (PhP, PhPlate AB, Stockholm, Sweden, www.phplate.se) (4). This system generates E. coli fingerprints by comparing their sugar fermentation capabilities while simultaneously determining resistance profiles to specific fixed-concentration antibiotics. The PhP method consistently identified two distinct ESBL-producing E. coli with unique fingerprints (PhP_1_ and PhP_2_) (Fig. 1). Subsequent multilocus sequence typing via whole-genome sequencing (WGS) revealed that the PhP_1_ and PhP_2_ isolates belonged to the clinically relevant clonal groups ST131 and ST648, respectively. However, a third fingerprint (PhP_3_) exhibiting multidrug resistance was found to be the most prevalent type in this data set. In this study, we have characterized the PhP_3_ isolates and compared their environmental survival and human colonization to the more well-known high-risk clones ST131 and ST648.

A total of 201 of 3,123 isolates (6.4%) had the PhP_3_ type, which was distinguishable from the other isolates by its ability to ferment lactose, rhamnose, and sorbose, with sucrose, raffinose, and gal-lactone fermentation being less efficient (Fig. 1). 11 representative PhP_3_ isolates that were obtained from 11 different sampling occasions in 10 of the study period’s 15 months (Fig. 1; Table S1) were sequenced using an Illumina MiSeq, following a previously described method (2). The sequencing data were assembled and annotated using BACTpipe v 2.7.0. (5), and the presence of antibiotic resistance genes, MLST profile, and plasmid typing were determined via an analysis using the CGE pipeline (6) (Table S1). All of the PhP_3_ isolates were identified as E. coli ST2797 and contained the IncFIA, IncH1A, and/or IncH1B, IncQ1, and ColRNAI plasmids as well as the resistance genes *sul2*, *dfrA7*, *bla_TEM-1B_*, and *tet*(B), thereby conferring resistance to sulfonamides, trimethoprim, ampicillin, and tetracycline, respectively. The MICs (7) of these strains confirmed the multidrug resistant (MDR) pattern (trimethoprim, >32 μg/mL; ampicillin, >128 μg/mL; tetracycline, >256 μg/mL).

ST2797 has not been extensively studied; only 11 isolates are described in Enterobase (8), and these were collected between 2010 and 2021 from human, wastewater, and environmental samples in Europe, the USA, Ecuador, Australia, and China. Two of those isolates reported ESBL genes (Table S2) (9–11). This suggests that ST2797 could spread and evolve across different niches. To investigate the evolutionary relationship between the ST2797 from this study and the eight ST2797 isolates from Enterobase that had available genome sequences, a phylogenetic tree was built, based on the core genome single nucleotide polymorphisms (cgSNPs). The analysis showed that our ST2797 isolates displayed genetic differences among themselves but were generally more closely related to each other than to the Enterobase ST2797 isolates. The BE10 and BE12 isolates were more diverse, as they were found in more than one subcluster. These two isolates were collected in December of 2016 and February of 2017, which were months in which the prevalence of ST2797 was high (Fig. S1). The observed genetic differences among the isolates suggest that the environment of the wastewater pump station may have facilitated further genetic changes over time.

To further characterize the genetic context of the antibiotic resistance genes (ARGs), contigs that were longer than 10 kb and contained ARGs were selected and aligned against the NCBI database. This revealed that all of the contigs were assigned to complete plasmid sequences of various Enterobacterales. Four plasmids with the highest identity (80 to 90%) and coverage (>70%) were identified, including the IncHI1 plasmids pB71 and pF8475 from environmental Salmonella Typhimurium isolates in the Czech Republic, the plasmid pSRC27-H from an equine Salmonella Typhimurium, and the IncF plasmid pME5 from an environmental E. coli isolate from the Seine estuary in France (Fig. 1C). These plasmids contained additional ARGs (catA1/2 and *aadA1*), but they lacked the *dfrA7* gene that was found in the ST2797 isolates of this study and was located next to a *qacE*Δ1 gene encoding resistance to disinfectants. In addition to *qacEΔ1*, *dfrA7* was also surrounded by the class 1 integron *intI1* and *sul1*. The presence of *intI1* serves as evidence of the horizontal gene transfer (HGT) of genes of clinical origin being widespread in the community, conferring adaptive advantages, and being associated with anthropogenic pollution that is likely present among bacteria from wastewater streams (12–14). All of our ST2797 plasmids carried the *tra* genes that are required for plasmid mobilization by HGT (15).

Other studies of antibiotic-resistant E. coli and other enterobacteria in wastewater treatment plants and the environment frequently identify certain clonal groups. For example, ST131, ST648, ST10, ST48, ST405, and ST38 are frequently detected and commonly reported to carry both ESBL-encoding genes and emerging novel resistance genes that encode the KPC and MCR-1 enzymes that confer resistance to carbapenems and colistin, respectively (16–18). The finding of ST2797 as the most prevalent ST in our data set indicates that there may be other, yet unrecognized, STs that are equally prevalent in the environment and are possibly involved in human and animal disease. The relatively high frequency in wastewater and the ability to pick up ESBL resistance are warning signs that ST2797 should be included in the screening of emerging antibiotic-resistant E. coli clones, globally.

**FIG 2:**
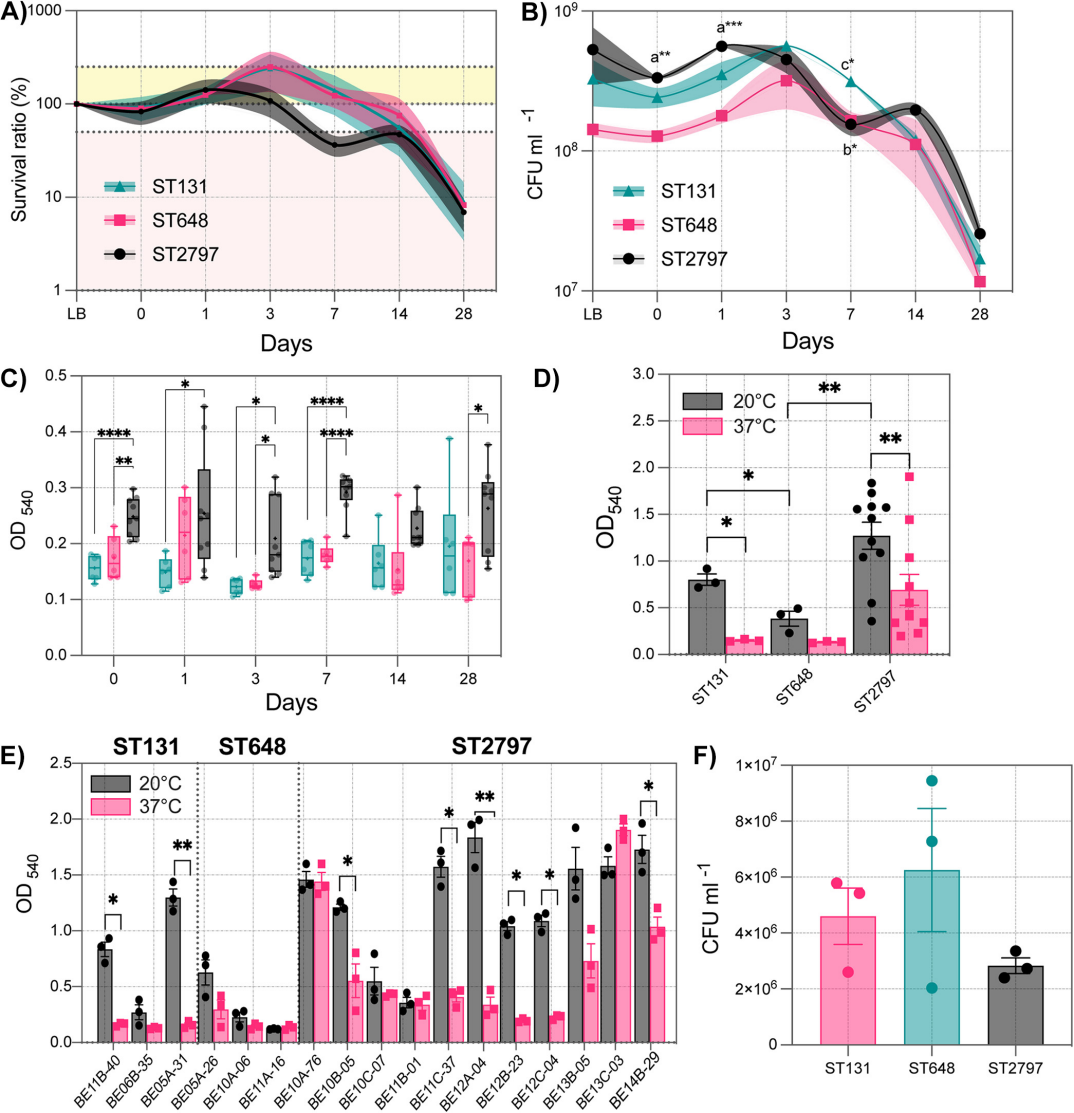
Bacterial survival, biofilm formation, and bacterial adherence to epithelial cells. (A) The survival ratio (%) and (B) the bacterial count (CFU/mL) of representative ST131 (*n* = 3), ST68 (*n* = 3), and ST2797 (*n* = 3 during incubation at 20°C in lake water microcosms [LWM]). Bacterial growth (>100%) and cell death (<50%) were colored yellow and red, respectively. The statistical analysis of panel B was performed using a repeated measures (RM) two-way analysis of variance (ANOVA) with Tukey’s multiple-comparison test. Comparisons that produced significant changes were coded as “a”, “b”, and “c” in panel B, and they correspond to ST2797 versus ST648, ST2797 versus ST131, and ST648 versus ST131, respectively. The error bars show the standard error of the mean of 3 independent experiments. (C) The quantification and comparison of surface-adhered biofilms in ST131 (red), ST648 (green), and ST2797 (black) isolates after 1, 2, 3, 7, 14, and 28 days of incubation in LWM at 20°C. Six independent CV experiments were performed, and the average values are plotted along with the standard deviations (error bars). The *P* values were determined via a one-way analysis of variance (ANOVA) with Tukey’s multiple-comparison test. (D) The quantification and comparison of the surface-adhered biofilms in ST131 (*n* = 3), ST648 (*n* = 3), and ST2797 (*n* = 11) after 48 h of incubation in rich medium (LB) at 20°C or 37°C. (E) The biofilm production of each isolate was measured to characterize the within-strain variability in the production of biofilm in response to temperature. Three independent CV experiments were performed, and the average values are plotted along with the standard deviations (error bars). The *P* values (*, *P* < 0.05; **, *P* < 0.01; ***, *P* < 0.001; ****, *P* < 0.0001) were determined via *t* tests. (F) Adherence assays were performed for 5 h in DMEM with 10% FBS. The numbers of bacterial cells (CFU/mL) adhered to HT-29/Caco-2 cell monolayers for the ST131, ST648, and ST2797 strains. The assays were performed in triplicate, and the graphs show the mean and standard deviation (error bars). The statistical analysis was performed using a one-way analysis of variance (ANOVA), but no significant differences were found.

Given that the ST2797 isolates, together with the ST131 and ST648 isolates (two high-risk clones of E. coli), were repeatedly isolated from a wastewater pump station, we evaluated the ability of these dominant circulating clones to survive in freshwater. For this experiment, we selected three representative isolates of each ST type and individually cultured them in 50 mL of sterile filtered lake water microcosms (LWM) at 20°C, as described elsewhere (19). The number of bacterial cells was recorded by counting the CFU in LB plates during growth in rich medium (LB broth as a control) immediately after the inoculation of the bacteria in LWM (time zero), and at 1, 3, 7, 14, and 28 days after inoculation (Fig. 2A). The three clones exhibited similar survival rates, and significant differences were observed on days 0 (ST2797 and ST648, *P* < 0.01), 1 (ST2797 and ST648, *P* < 0.001), and 7 (ST2797 and ST131, *P* < 0.05; ST648 and ST131, *P* < 0.05). ST648, ST131, and, to a lesser extent, ST2797 increased their bacterial numbers during the first 14 days of incubation. On day 28 of the experiment, only 8.2%, 5.2%, and 4.8% of the ST131, ST648, and ST2797, respectively, remained viable and culturable, meaning that ST2797 has a survival capacity that is comparable to those of ST648 and ST131 (Fig. 2A and B). Whether they share a similar molecular mechanism of adaptation to the freshwater environment warrants further study.

The persistence of bacteria within water distribution systems is associated with the ability of the bacteria to form biofilms (20). To evaluate the potential to produce biofilms during freshwater incubation, we set up a 96-well LWM that consisted of 100 μL aliquots of the LWM (time point 0) from the previous experiment that were inoculated into 96-well polystyrene microtiter plates. A 96-well plate per time point was inoculated, sealed, and incubated at 20°C. Biofilm formation was evaluated via crystal violet retention (21). As shown in Fig. 2C, at each time point, the ST2797 isolates showed a significantly higher number of bacteria attached to the abiotic surfaces of the plates than did the ST131 and ST648 isolates. None of the ST clones showed a progressive increase in biofilm formation.

Given that the surviving freshwater E. coli population of ST2797 may use biofilms to persist in the wastewater environment and that this bacterial population can potentially colonize humans, we wanted to test whether mammalian intestinal conditions, such as 37°C and nutrient-rich environments, also induce biofilm formation in ST2797 and the high-risk clones ST131 and ST648. As shown in Fig. 2D, biofilm production was higher for all isolates at 20°C, compared to 37°C. At 20°C, ST2797 produced 1.5-fold and 4-fold (*P* < 0.01) more biofilm than did ST131 and ST648, respectively. In concordance with our previous findings (2), ST131 produced more biofilm than did ST648 at 20°C. We also observed that 20°C induced higher biofilm production than did 28°C (2). As shown in Fig. 2E, a large amount of variability in biofilm production was observed within ST2797. Specifically, out of the 11 ST2797 isolates, 6 exhibited a temperature-dependent increase in biofilm production, whereas the remaining isolates showed similar values at both temperatures. Due to the protective nature of the biofilm, the results suggest that the ST2797 isolates can be fitter at lower temperatures than can the ST131 and ST648 isolates. The results also suggest that some ST2797 that are strong biofilm producers might persist longer under these environmental conditions, particularly when nutrients are available.

Pandemic E. coli ST131 and ST648 are well-known to colonize the human gastrointestinal tract or urinary tract and cause infections (22, 23). To test whether ST2797 differs in its ability to adhere to human epithelial cells, compared to ST131 and ST648, *in vitro* coculture experiments with bacterial isolates and monolayers of HT-29/Caco-2 cells were performed. The results showed no significant differences in the numbers of attached bacteria after infection among the different STs. However, ST648 exhibited an elevated/increased binding phenotype, which was followed by those of ST131 and ST2797 (Fig. 2F). These findings suggest that ST2797 may have a similar potential to colonize the human intestinal tract.

In conclusion, this is the first report to describe the emergence as well as the genomic and phenotypic features of multidrug resistant E. coli from the ST2797 clonal group. The bacteria from this sequence type were the most prevalent bacteria that were isolated from a community wastewater pump station, and they displayed similar phenotypic traits to the pandemic E. coli sequence types 131 and 648. These findings prompt further research on the prevalence and dissemination of emerging wastewater clones and raise concerns about waterborne transmission to the community.

### Data availability.

Draft genomes of E. coli ST2797 were deposited into the NCBI database under BioProject PRJNA527183.
